# A Case of Aneurism of the Aorta Opening into the Trachea

**Published:** 1874-07

**Authors:** L. S. McMurtry

**Affiliations:** New Orleans


### Miscellaneous.

### A Case of Aneurism of the Aorta, Opening into Trachea, and Producing Instant Death, with Pathological Specimen.

By L. S. McMurtry, M. D., New Orleans.

C. C., aet. 34, laborer, of robust appearance, was admitted to ward 19, Charity Hospital, February 25, 1874. On examination patient stated, that he had always experienced good health until a few weeks past, when he began to suffer with a cough and other symptoms which he referred to the throat; also stated that the symptoms had become more urgent of late, breathing more difficult, and that he had several times suffered with violent paroxysms of coughing, in each of which he thought that he would be suffocated; these paroxysms of coughing were of more frequent occurrence lately. Patient had a very anxious expression of countenance; breathed with the mouth open, and with every act of respiration loud tracheal sounds could be heard at a distance from the patient. Voice normal; pulse good, regular at 82 per minute. Temperature normal; appetite good; urine normal. No abnormality detected by careful inspection and percussion of the chest. Line of cardiac dulness and apex beat in normal situation. On auscultation, the tracheal and bronchial sounds were so loud as to obscure every other sound. For this reason the heart sounds could not be well discerned, but the valves could be heard to close, and no abnormality could be discerned. No bruit could be heard along the course of the great vessels of the neck and thorax, the tracheal and bronchial sounds being so loud as to drown every other sound. On the day after the patient was admitted, I made a careful examination with the laryngyscope, and could detect nothing abnormal beyond slight congestion of the mucous membrane of the parts.

The diagnosis at this time was not positive, and I attributed the symptoms either to some growth in the trachea directly obstructing the tube, or to an aneurism or other tumor pressing upon the trachea and thus diminishing the calibre of the tube.

The equable condition of the circulation, the vigor of the patient (not having reached that age when degenerative changes in the arterial coats usually occur,) the natural voice, and the fact that no bruit could be heard rising above the turbulent bronchial and tracheal sounds—all rather inclined me to believe that there was some growth directly obstructing the trachea. A tonic, and a simple cough mixture were prescribed. The patient remained in almost the same condition for several weeks, continually suffering with dyspnœa, and violent paroxysms of coughing with a feeling of impending suffocation. The cough was accompanied with slight mucous expectoration, and after coughing violently for several minutes he frequently would expectorate a small quantity of blood unmixed with air. This evidently came from the engorged mucous membrane. The treatment consisted of tonics, with remedies to allay the irritability of the air tract. Patient was not confined to bed, and his general condition continued good. On March 16, a talented physician and skillful diagnostician examined the case with me. He, too, rather preferred to believe that there was some growth in the trachea than that an aneurism was the cause of the trouble. No change was made in the treatment. On the morning of the 18th, patient was as well as usual, and going about the ward. In the evening he was walking about the grounds, and on returning to the ward, having ascended a flight of steps, he was seized with a violent paroxysm of coughing, and began to bleed furiously. The blood poured from the mouth and nose, of bright arterial hue, and death occurred at once. Autopsy twelve hours after death.

Blood poured from mouth and nose on placing the body on the table. Lungs typically healthy, but colored by the blood which had flooded the air cells. Stomach filled with blood; heart normal. The specimen herewith presented shows the cause of the symptoms and death. An aneurism of considerable size is situated upon the descending portion of the arch of the aorta, which has pressed upon the trachea, and by inflammation become adherent to the same, and an opening of large size may be seen where the sac communicated with the tube, thus explaining the fatal hemorrhage. It will be observed that the coats of the artery have undergone degenerative change within and in the vicinity of the sac. No degeneration in the arterial coats was discovered elsewhere. Other organs of body normal in appearance.

This case, gentlemen, has many points of interest, but illustrates particularly the fact that very extensive lesions may produce symptoms of such ambiguous nature as to render a positive diagnosis almost impossible. In this case, also, the question was considered as to whether or not it would not be proper to perform tracheotomy if the difficult breathing and violent coughing should become more urgent. In fact since the case terminated, a medical teacher of ability and experience has informed me that in a very similar case he opened the trachea, the patient died from hemorrhage, and the autopsy revealed a condition almost identical with that of the case here presented. *Proceedings N. O. Med. Association. New Orleans Med. Journal.*

